# Surgeon decision-making and implant selection in primary total knee arthroplasty: association of training, experience, and robotic-assisted surgical innovation with implant selection

**DOI:** 10.3389/fmed.2026.1834294

**Published:** 2026-05-29

**Authors:** Ali Ibrahim Alhefzi, Abdullah Raizah, Shaker Hassan S. Alshehri, Fareed F. Alfaya, Ghada Mohamed Koura, Ahmed Mohamed Elshiwi, Ajay Prashad Gautam, Saleh M. Kardm, Ravi Shankar Reddy

**Affiliations:** 1 Department of Orthopedic Surgery, College of Medicine, King Khalid University, Abha, Saudi Arabia; 2 Department of Medical Rehabilitation Sciences, College of Applied Medical Sciences, King Khalid University, Abha, Saudi Arabia; 3 Department of Physical Therapy, Saudi German Hospital, Aseer, Saudi Arabia; 4 Department of Surgery, College of Medicine, Najran University, Najran, Saudi Arabia

**Keywords:** arthroplasty, knee, prosthesis design, replacement, robotic surgical procedures

## Abstract

**Introduction:**

Implant selection in primary total knee arthroplasty (TKA) remains a critical yet variable aspect of surgical decision-making, associated with both patient characteristics and surgeon-related factors. With the increasing integration of surgical innovations such as robotic-assisted techniques, understanding contemporary determinants of implant choice has become essential. This study aimed to evaluate implant selection patterns and identify independent factors associated with the use of cruciate-retaining (CR) vs. posterior-stabilized (PS) implants in a modern clinical setting. This study was designed as a retrospective observational analytical study, with data analyzed within a cross-sectional framework.

**Methods:**

A cross-sectional study was conducted on 280 patients undergoing primary TKA at a high-volume tertiary care center between May 2024 and April 2025. Data on patient demographics, clinical characteristics, and implant-related variables were collected. Surgeon-level factors, including fellowship training, years in practice, and use of robotic or navigation systems, were also analyzed. Multivariate logistic regression was performed to identify independent factors associated with the CR implant selection.

**Results:**

CR implants were used in 60.0% of cases, whereas PS implants were used in 40.0% (*p* = 0.017). Cemented fixation predominated (75.0%, *p* = 0.004). Fellowship-trained surgeons and those with fewer years in practice demonstrated higher CR utilization (*p* = 0.019 and *p* = 0.041, respectively). Multivariable analysis identified fellowship training (OR 2.14, *p* = 0.002), ≥10 years in practice (OR 1.45, *p* = 0.027), and robotic assistance (OR 1.61, *p* = 0.012) as independent factors associated with CR selection. Increasing age (OR 1.02, *p* = 0.045) and male sex (OR 1.35, *p* = 0.042) were also associated with CR use, whereas BMI, ASA class, and navigation were not.

**Conclusion:**

Implant selection in primary TKA is influenced by a combination of surgeon training and experience, patient characteristics, and the adoption of surgical technologies such as robotic assistance. These findings highlight the impact of surgical innovation on decision-making in modern arthroplasty practice and underscore the need for standardized, evidence-based approaches to reduce variability in implant selection.

## 1 Introduction

Total knee arthroplasty (TKA) is one of the most commonly performed orthopedic procedures worldwide and is widely regarded as a highly effective surgical intervention for relieving pain and restoring function in patients with advanced knee osteoarthritis (1). With increasing life expectancy and demand for joint preservation among active older adults, the global burden of primary TKA is projected to rise substantially in the coming decades (2). Although the procedure demonstrates excellent long-term survivorship, functional outcomes and complication rates remain variably associated with implant selection and surgical technique (3). Among the key decisions facing orthopedic surgeons during TKA is the choice of implant design—particularly between cruciate-retaining (CR) and posterior-stabilized (PS) prostheses—as well as fixation method, bearing surface, and constraint level (4). These choices must balance biomechanical considerations, surgeon familiarity, and patient-specific anatomy (4). Despite extensive research on implant longevity and kinematic performance, considerable variability exists in clinical practice regarding how implants are selected, particularly in the absence of clearly superior outcomes for one design over another (5).

Previous literature has explored outcomes associated with various implant types, yet findings have been heterogeneous (6). Studies by Steinert et al. (7) and Kalaai et al. (8) reported comparable implant survivorship between CR and PS designs over 15–20 years, with CR implants potentially offering improved proprioception and better replication of native knee kinematics. Movassaghi et al. (4) found that CR designs were more likely to be used in patients with less deformity and an intact posterior cruciate ligament. Meanwhile, cemented fixation remains the gold standard based on long-term registry data, although cementless designs have gained renewed interest, particularly in younger or high-demand patients, as noted by Alzarooni et al. (9) and Lauck et al. (10). Additionally, recent studies suggest that surgeon-related variables, such as fellowship training and operative volume, and access to surgical technologies, such as robotics and navigation, may influence implant choice (11). However, there remains limited evidence on how surgeon- and patient-level factors interact to guide implant decision-making in real-world clinical practice (11).

There is a growing need to better understand the determinants of implant selection in TKA, particularly within the context of modern arthroplasty techniques and evolving implant technologies (12). While previous research has largely focused on clinical outcomes or registry trends, few studies have comprehensively assessed how individual surgeon training, experience, and case volume influence implant-related choices at the point of care (13). Furthermore, the extent to which patient demographic and radiographic factors contribute to these decisions within a standardized institutional environment remains insufficiently defined (14). Clarifying these influences is critical for reducing practice variability, enhancing surgical education, and informing evidence-based guidelines (14).

The objective of this study was to evaluate implant selection patterns in primary TKA at a high-volume tertiary care center and to identify both surgeon- and patient-level factors independently associated with the use of CR vs. PS implants. We hypothesized that fellowship training in arthroplasty, fewer years in practice, and the use of intraoperative technology would be positively associated with CR implant selection. At the same time, patient characteristics such as younger age and lower body mass index (BMI) were associated with increased use of CR implants. However, the present findings demonstrate that this relationship is sensitive to analytical approach. Although unadjusted comparisons suggested greater CR/CRT use among younger patients, the adjusted analysis suggested a slight increase in the likelihood of CR selection with advancing age. This divergence is likely attributable to confounding factors included in the regression model and to the treatment of age as a continuous variable, underscoring the importance of interpreting adjusted estimates when assessing independent predictors.

## 2 Methods

### 2.1 Study design, ethics, and settings

This cross-sectional study was conducted between 12 May 2024 and 10 April 2025 at the Arthroplasty Clinic, Department of Orthopedic Surgery, College of Medical Sciences, King Khalid University, Kingdom of Saudi Arabia. Ethical approval was obtained from the Institutional Review Board of King Khalid University (Approval Number: KKU-163-2025-31), and written informed consent was obtained from all participants. The study adhered strictly to the ethical standards outlined in the Declaration of Helsinki. Written informed consent included consent for the use of de-identified data for research purposes in accordance with institutional ethics approval. All data made available through the Zenodo repository were fully de-identified prior to deposition, contain no direct personal identifiers, and were shared in a manner consistent with ethics approval and applicable local institutional regulatory requirements governing human participant research and data privacy. This study represents a retrospective observational analytical design based on a consecutive series of surgical cases collected over a defined study period. Although analyzed in a cross-sectional framework, the data incorporate temporally linked patient characteristics, surgeon decision-making, and intraoperative variables recorded at the time of the procedure.

### 2.2 Participants

Participants were recruited through consecutive sampling from patients scheduled for primary total knee arthroplasty at the Arthroplasty Clinic of King Khalid University Hospital during the study period. Eligible individuals were identified from outpatient arthroplasty referral lists and screened through clinical consultations and preoperative evaluations. The diagnosis of advanced knee osteoarthritis was confirmed using a combination of clinical criteria—persistent pain, reduced joint function, and failure of conservative management—and radiographic evidence consistent with Kellgren-Lawrence grade 3 or 4 changes (15).

Inclusion criteria comprised adults aged 50 years or older undergoing elective, unilateral, primary TKA for a confirmed diagnosis of primary osteoarthritis, post-traumatic arthritis, or inflammatory arthritis. All participants were required to have completed preoperative imaging and to be deemed medically fit for surgery, classified as American Society of Anesthesiologists (ASA) physical status I-III (15). Exclusion criteria included patients undergoing revision TKA, undergoing simultaneous bilateral procedures, or having prior ipsilateral knee arthroplasty or a major deformity requiring a constrained or hinged prosthesis. Additional exclusions were made for patients with incomplete perioperative records or who declined to provide informed consent. All participants underwent baseline demographic and clinical assessments, including age, sex, BMI, comorbidity evaluation, and preoperative imaging review, to confirm eligibility prior to enrollment.

### 2.3 Implant type

Implant type was classified as either cruciate-retaining (CR) or posterior-stabilized (PS), based on the intraoperative decision documented in the operative record (16). CR implants preserve the posterior cruciate ligament and were selected when intraoperative assessment confirmed adequate ligament integrity and favorable femoral-tibial alignment (16). PS implants, which include a central post and cam mechanism to substitute for the function of the posterior cruciate ligament, were used in cases of posterior cruciate insufficiency, deformity, or instability (16). Selection was made at the attending surgeon's discretion and recorded in the standardized surgical template (16). Classification was verified from implant catalog numbers and surgical logs.

### 2.4 Fixation method

Fixation was defined as either cemented or cementless, based on the method used for placement of the femoral and tibial components, as documented in the operative note and implant registry (16). Cemented fixation involved the application of polymethylmethacrylate (PMMA) bone cement to secure the components to the bone surfaces. Cementless fixation relied on press-fit techniques with porous-coated or hydroxyapatite-coated surfaces to allow osseointegration (17). The choice of fixation method was guided by intraoperative bone quality and surgeon preference, and verification was performed by cross-checking implant labels and operative records (17).

### 2.5 Bearing surface

Bearing surface was recorded as either highly cross-linked polyethylene (HXLPE) or conventional polyethylene, based on implant manufacturer specifications documented in operative inventory sheets (18). HXLPE components underwent additional irradiation and thermal processing to improve wear resistance, while conventional polyethylene did not. All implant inserts were identified using catalog numbers and manufacturer documentation to ensure accurate classification (1).

### 2.6 Constraint level

Constraint level was recorded as either standard (non-constrained) or varus-valgus constrained (VVC), based on the implant's mechanical design and intraoperative use (19). Standard implants allowed physiologic varus-valgus motion and rotational freedom, whereas VVC components provided increased mediolateral stability through a larger post and deeper tibial cam articulation (19). Constraint level was determined based on the final implant construct used in each case, confirmed through operative notes and implant catalog data.

### 2.7 Use of robotic assistance

Robotic assistance was defined as the intraoperative use of a robotic surgical system for bone preparation and implant positioning. In this study, the MAKO robotic-arm system (Stryker, USA) was used in all applicable cases (20, 21). The system required preoperative CT-based planning and intraoperative haptic guidance. Surgeons followed standard calibration and registration protocols, including precise anatomic landmark mapping and verification of bone cuts (21). All procedures using robotic assistance were documented in the surgical record, and utilization was verified by cross-referencing the operative schedule and robotic system logs (21). Use of MAKO robotics and OrthoPilot navigation was based on surgeon preference, technology availability, and case-specific intraoperative planning rather than a standardized protocol mandating implant selection. The use of technology was intended to assist with component positioning, alignment strategy, and soft-tissue balancing, but did not independently determine the selection of cruciate-retaining or posterior-stabilized implants. Potential learning-curve effects and differences in case selection may have influenced technology use and are acknowledged as possible sources of selection bias.

### 2.8 Use of navigation

Computer-assisted navigation was defined as the intraoperative use of an infrared-based navigation system (OrthoPilot, B. Braun Aesculap) to guide bone alignment and implant positioning (22). The navigation system utilized pre-calibrated trackers attached to the femur and tibia and required surface mapping and registration of key anatomic landmarks. Navigated procedures followed standardized workflows, and verified alignment per the system's manufacturer guidelines (22). Cases were classified as navigation-assisted if real-time intraoperative alignment feedback was used during any stage of the procedure.

### 2.9 Age

Age was recorded in years at the time of surgery, as documented in the hospital's electronic medical record. It was treated as a continuous variable in statistical analyses. Age was included as a key demographic factor due to its known association with implant selection, bone quality, and functional outcomes in TKA, as supported by registry-based and prospective studies.

### 2.10 Sex

Sex was recorded as male or female, based on the patient's demographic information at the time of enrollment. This binary variable was used in analyses to evaluate sex-based differences in implant selection and is routinely collected as a demographic parameter in arthroplasty studies.

### 2.11 BMI

BMI was calculated as weight in kilograms divided by height in meters squared (kg/m^2^) from preoperative anthropometric measurements recorded during the anesthesia pre-assessment clinic visit. BMI was analyzed both as a continuous variable and as a dichotomous variable (< 30 kg/m^2^ vs. ≥30 kg/m^2^). Measurements were performed by trained nursing staff using calibrated digital scales and stadiometers.

### 2.12 ASA physical status classification

ASA class was determined preoperatively by the attending anesthesiologist based on the American Society of Anesthesiologists physical status classification system (23). Patients were grouped into Class I (healthy), Class II (mild systemic disease), or Class III (severe systemic disease). ASA classification was recorded directly from the anesthesia assessment form and was used to assess baseline surgical risk.

### 2.13 Diagnosis

Preoperative diagnosis was categorized into primary osteoarthritis, post-traumatic arthritis, or inflammatory arthritis, as documented in the pre-surgical evaluation by the treating orthopedic surgeon. Diagnosis was based on clinical history, physical examination, and radiographic findings. Supporting diagnostic data included joint space narrowing, osteophyte formation, or a history of trauma or autoimmune joint disease. This variable was extracted from the standardized preoperative assessment form and cross-checked against radiologic reports to ensure accuracy.

### 2.14 Kellgren-Lawrence (KL) grade

Radiographic severity of knee osteoarthritis was assessed using the Kellgren-Lawrence (KL) grading scale based on preoperative anteroposterior standing knee radiographs (24). Grading was performed by the attending orthopedic surgeon and confirmed by a musculoskeletal radiologist. Only grades 3 and 4 were included in this study, corresponding to moderate and severe OA, respectively (24). Grade 3 was defined by moderate joint space narrowing and definite osteophyte formation, while grade 4 was characterized by marked joint space loss, sclerosis, and deformity.

### 2.15 Laterality

Laterality referred to the side of the operated knee—either right or left—and was documented from operative records. This variable was included to assess potential unintentional biases in surgical technique or implant selection associated with dominant-side procedures, although it was not hypothesized to affect outcomes.

### 2.16 Patellar resurfacing

Patellar resurfacing status was recorded as “Yes” or “No” based on intraoperative decisions documented in the surgical note. Resurfacing involved replacing the native patellar surface with a polyethylene button and was performed at the surgeon's discretion, based on cartilage wear, patellar tracking, and component compatibility. Verification was conducted using implant logs and operative records.

### 2.17 Sample size calculation

The sample size was calculated using G^*^Power version 3.1.9.7 based on a chi-square test to detect an association between surgeon-level characteristics and implant selection patterns in primary total knee arthroplasty. A moderate effect size (w = 0.3) was assumed, based on a conventional effect size commonly used when prior study-specific estimates are limited and consistent with previous studies examining categorical clinical decision-making. The alpha level was set at 0.05 (two-tailed), with 80% power to minimize the risk of Type II error, and a 1:1 allocation ratio was used to reflect an even distribution across implant-type categories. Based on these parameters, the minimum required sample size was 264. To account for potential data exclusions or incomplete records, the final sample size was set at 280 cases, which provided adequate power for subgroup analyses and multivariable regression. Model stability was further assessed using an events-per-variable approach for the multivariable logistic regression. With 168 cruciate-retaining events and 9 independent variables included in the model, the events-per-variable ratio was 18.7, exceeding commonly accepted minimum thresholds for regression adequacy.

### 2.18 Data analysis

All statistical analyses were performed using IBM SPSS Statistics version 24.0 (IBM Corp., Armonk, NY, USA). Data were assessed for normality using visual inspection of histograms and the Shapiro-Wilk test, and all continuous variables were confirmed to follow a normal distribution. As such, parametric statistical methods were applied throughout. Descriptive statistics were used to summarize participant characteristics, with continuous variables reported as means and standard deviations (SDs) and categorical variables as frequencies and percentages. Independent-samples *t*-tests were used to compare means between two groups (e.g., CR vs. PS implant recipients), while chi-square tests were used to evaluate associations between categorical variables, such as implant type and fixation method. Pearson correlation coefficients were calculated to assess linear associations between continuous variables, including age, BMI, and implant selection metrics. To identify independent factors of cruciate-retaining implant selection, multivariate logistic regression analysis was conducted, adjusting for relevant patient and surgeon-level covariates. Model outputs included odds ratios (ORs) with corresponding 95% confidence intervals (CIs). All statistical tests were two-tailed, and a *p*-value of less than 0.05 was considered statistically significant. Collinearity among independent variables was assessed before model estimation using variance inflation factors, and no evidence of problematic multicollinearity was identified. Exploratory interaction testing between years in practice and robotic assistance did not materially alter the model estimates and was therefore not retained in the final model.

## 3 Results

The study population had a mean age of 67.43 ± 8.91 years and a mean BMI of 29.76 ± 4.82 kg/m^2^, with a female predominance (57.14%) (Table 1). The majority of patients were classified as ASA II (62.14%), with fewer in ASA I (10.00%) or III (27.86%). Most procedures were performed for primary osteoarthritis (85.00%), and more than half of the knees had Kellgren-Lawrence Grade 4 changes (55.71%). Right-sided procedures slightly predominated (56.43%). Cruciate-retaining implants were used in 60.00% of cases, while 40.00% received posterior-stabilized implants. Cemented fixation was used in 75.00% of patients, compared with 25.00% who received cementless implants. Navigation was used in 33.57% of cases, and robotic assistance in 18.57%, reflecting selective adoption of technology across the cohort.

**Table 1:** Demographic and clinical characteristics of the study population (*n* = 280).

Variable	Total (*n* = 280)
Age (years)	67.43 ± 8.91
Sex (male)	120 (42.86%)
Sex (female)	160 (57.14%)
Body mass index (kg/m^2^)	29.76 ± 4.82
ASA class I	28 (10.00%)
ASA class II	174 (62.14%)
ASA class III	78 (27.86%)
Laterality (right)	158 (56.43%)
Laterality (left)	122 (43.57%)
Diagnosis: primary osteoarthritis	238 (85.00%)
Diagnosis: post-traumatic arthritis	24 (8.57%)
Diagnosis: inflammatory arthritis	18 (6.43%)
Preoperative Kellgren-Lawrence Grade 3	124 (44.29%)
Grade 4	156 (55.71%)
Cruciate-retaining implant	168 (60.00%)
Posterior-stabilized implant	112 (40.00%)
Cemented fixation	210 (75.00%)
Cementless fixation	70 (25.00%)
Use of navigation	94 (33.57%)
Use of robotic assistance	52 (18.57%)

ASA, American Society of Anesthesiologists; SD, standard deviation; BMI, body mass index; TKA, total knee arthroplasty.

Implant selection and surgical techniques demonstrated a clear predominance of conventional approaches (Table 2). Cruciate-retaining implants were used more frequently than posterior-stabilized designs (60.00% vs. 40.00%), with a strong preference for cemented fixation (75.00% vs. 25.00%). Highly cross-linked polyethylene was utilized in the majority of cases (82.14%), and standard constraint implants accounted for nearly all procedures (90.71%), indicating limited use of constrained designs (9.29%). Patellar resurfacing was performed in a substantial proportion of cases (69.29%), while navigation-assisted surgery was used in approximately one-third of procedures (33.57%). Robotic assistance was less commonly employed, accounting for only 18.57% of cases, reflecting lower adoption than other technological adjuncts.

**Table 2:** Implant characteristics and surgical variables (descriptive summary).

Variable	Category	*n* (%)
Implant type	Cruciate-retaining (CR)	168 (60.00)
	Posterior-stabilized (PS)	112 (40.00)
Fixation method	Cemented	210 (75.00)
	Cementless	70 (25.00)
Bearing surface	Highly cross-linked polyethylene	230 (82.14)
	Conventional polyethylene	50 (17.86)
Constraint level	Standard	254 (90.71)
	Constrained	26 (9.29)
Patellar resurfacing	Yes	194 (69.29)
	No	86 (30.71)
Navigation use	Yes	94 (33.57)
	No	186 (66.43)
Robotic assistance	Yes	52 (18.57)
	No	228 (81.43)

CR, cruciate-retaining; PS, posterior-stabilized. Values are presented as counts with percentages in parentheses.

**Figure 1:**
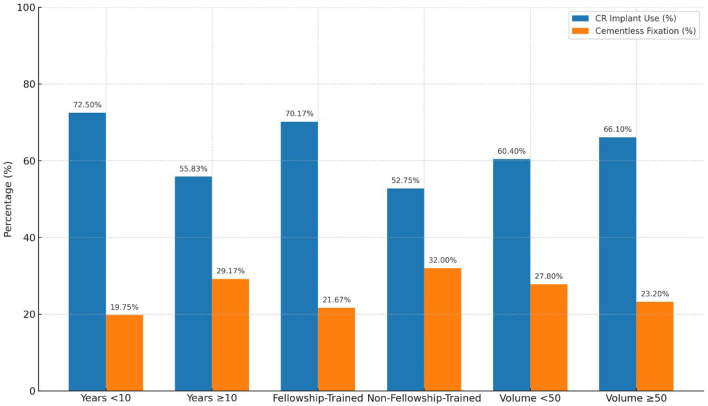
Cruciate-retaining implant use and cementless fixation rates stratified by surgeon experience, fellowship training, and case volume (*n* = 10).

Surgeons with fewer than 10 years in practice demonstrated significantly greater use of cruciate-retaining implants compared to those with ≥10 years (72.50% ± 5.32 vs. 55.83% ± 6.12, *p* = 0.041), along with a lower reliance on cementless fixation (19.75% ± 4.10 vs. 29.17% ± 4.80, *p* = 0.047) (Table 3 and Figure 1). Similarly, arthroplasty fellowship-trained surgeons suggested a higher preference for cruciate-retaining implants (70.17% ± 4.89 vs 52.75% ± 5.68, *p* = 0.019) and lower cementless use (21.67% ± 4.23 vs. 32.00% ± 4.45, *p* = 0.033) compared to non-fellowship-trained counterparts. Differences in implant selection and technology use by annual surgical volume were not statistically significant; surgeons performing ≥50 TKAs annually had comparable use of cruciate-retaining implants and cementless fixation compared with lower-volume surgeons (*p* = 0.326 and *p* = 0.412, respectively). In contrast, after adjustment in the multivariable model (Table 4), surgeons with ≥10 years in practice demonstrated higher odds of CR selection (OR 1.45). This reversal reflects confounding introduced by covariates included in the multivariable model, particularly surgeon age, practice setting, and operative volume, which are associated with both years in practice and implant selection.

**Table 3:** Surgeon-level characteristics and implant preferences (*n* = 10 surgeons).

Surgeon characteristic	Number of surgeons (*n*)	Mean CR implant use (%) ±SD	Mean PS implant use (%) ±SD	Mean cemented use (%) ±SD	Mean cementless use (%) ±SD	Mean use of robotics (%) ±SD	Mean use of navigation (%) ±SD	*p*-value (CR use)	*p*-value (Cementless use)
Years in practice: < 10 years	4	72.50 ± 5.32	27.50 ± 5.32	80.25 ± 4.10	19.75 ± 4.10	22.50 ± 3.75	30.25 ± 4.85	0.041	0.047
Years in practice: ≥10 years	6	55.83 ± 6.12	44.17 ± 6.12	70.83 ± 4.80	29.17 ± 4.80	14.67 ± 2.90	27.83 ± 5.10	0.041	0.047
Fellowship-trained in arthroplasty	6	70.17 ± 4.89	29.83 ± 4.89	78.33 ± 4.23	21.67 ± 4.23	24.00 ± 3.20	32.50 ± 4.60	0.019	0.033
Non-fellowship-trained	4	52.75 ± 5.68	47.25 ± 5.68	68.00 ± 4.45	32.00 ± 4.45	12.25 ± 2.70	24.00 ± 5.00	0.019	0.033
Annual TKA volume per surgeon: < 50	5	60.40 ± 6.20	39.60 ± 6.20	72.20 ± 5.00	27.80 ± 5.00	18.40 ± 3.85	25.20 ± 4.90	0.326	0.412
Annual TKA volume per surgeon: ≥50	5	66.10 ± 4.75	33.90 ± 4.75	76.80 ± 4.75	23.20 ± 4.75	19.20 ± 3.50	32.80 ± 4.35	0.326	0.412

CR, cruciate-retaining; PS, posterior-stabilized; SD, standard deviation; TKA, total knee arthroplasty.

**Table 4:** Multivariate logistic regression: factors associated with cruciate-retaining vs. posterior-stabilized implant choice.

Predictor variable	Odds ratio (OR)	95% confidence interval	*p*-value
Age (per year increase)	1.02	1.00–1.04	0.045
Sex (male vs. female)	1.35	1.01–1.81	0.042
BMI (per unit increase)	0.97	0.93–1.00	0.084
ASA class III (vs. I–II)	0.76	0.55–1.04	0.078
Fellowship-trained surgeon (yes vs. no)	2.14	1.32–3.47	0.002
Years in practice (≥10 vs. < 10)	1.45	1.05–2.01	0.027
Annual TKA volume (≥50 vs. < 50)	1.22	0.88–1.69	0.221
Use of robotics (yes vs. no)	1.61	1.11–2.34	0.012
Use of navigation (yes vs. no)	1.08	0.76–1.54	0.612

OR, odds ratio; CI, confidence interval; BMI, body mass index; ASA, American Society of Anesthesiologists; TKA, total knee arthroplasty; CR, cruciate-retaining; PS, posterior-stabilized.

**Figure 2:**
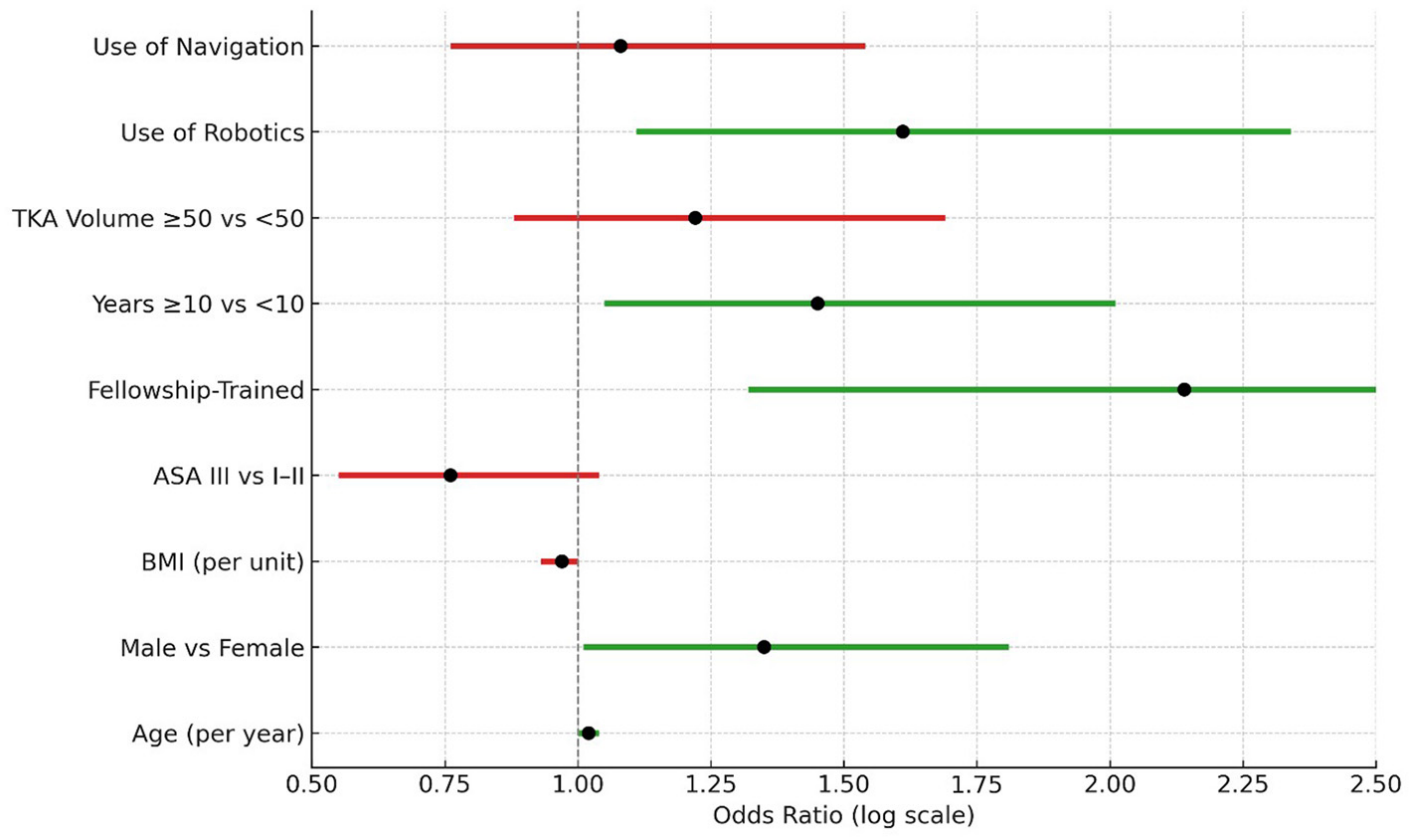
Multivariate logistic regression identifying independent factors of cruciate-retaining implant selection in total knee arthroplasty.

Multivariate logistic regression analysis identified several independent factors of cruciate-retaining implant selection over posterior-stabilized designs (Table 4 and Figure 2). Increasing age was associated with higher odds of CR implant use (OR 1.02, 95% CI 1.00–1.04, *p* = 0.045), as was male sex (OR 1.35, 95% CI 1.01–1.81, *p* = 0.042). While unadjusted analyses suggested greater CR use among patients aged < 65 years, the multivariable model treated age as a continuous variable and adjusted for relevant covariates. The observed positive association with increasing age, therefore, reflects model specification and confounding rather than a true contradiction between analyses. Fellowship training in arthroplasty suggested a strong and statistically significant association with CR preference (OR 2.14, 95% CI 1.32–3.47, *p* = 0.002), as did greater years in practice (OR 1.45, 95% CI 1.05–2.01, *p* = 0.027). Use of robotic assistance was also significantly associated with CR implant selection (OR 1.61, 95% CI 1.11–2.34, *p* = 0.012). Body mass index, ASA class III, annual case volume, and use of navigation were not independently associated with implant choice in this model.

The apparent difference between the descriptive findings in Table 3 and the adjusted odds ratio in Table 4 likely reflects confounding among surgeon-level variables and the distinction between unadjusted subgroup proportions and covariate-adjusted regression estimates. Accordingly, the multivariable model should be interpreted as estimating the independent association between years in practice and the outcome after adjustment for other included covariates. Model performance was additionally assessed using the Hosmer-Lemeshow goodness-of-fit test and model discrimination using the area under the receiver operating characteristic curve (AUC). The model demonstrated acceptable calibration (Hosmer–Lemeshow *p* = 0.42) and acceptable discrimination (AUC = 0.76).

**Figure 3:**
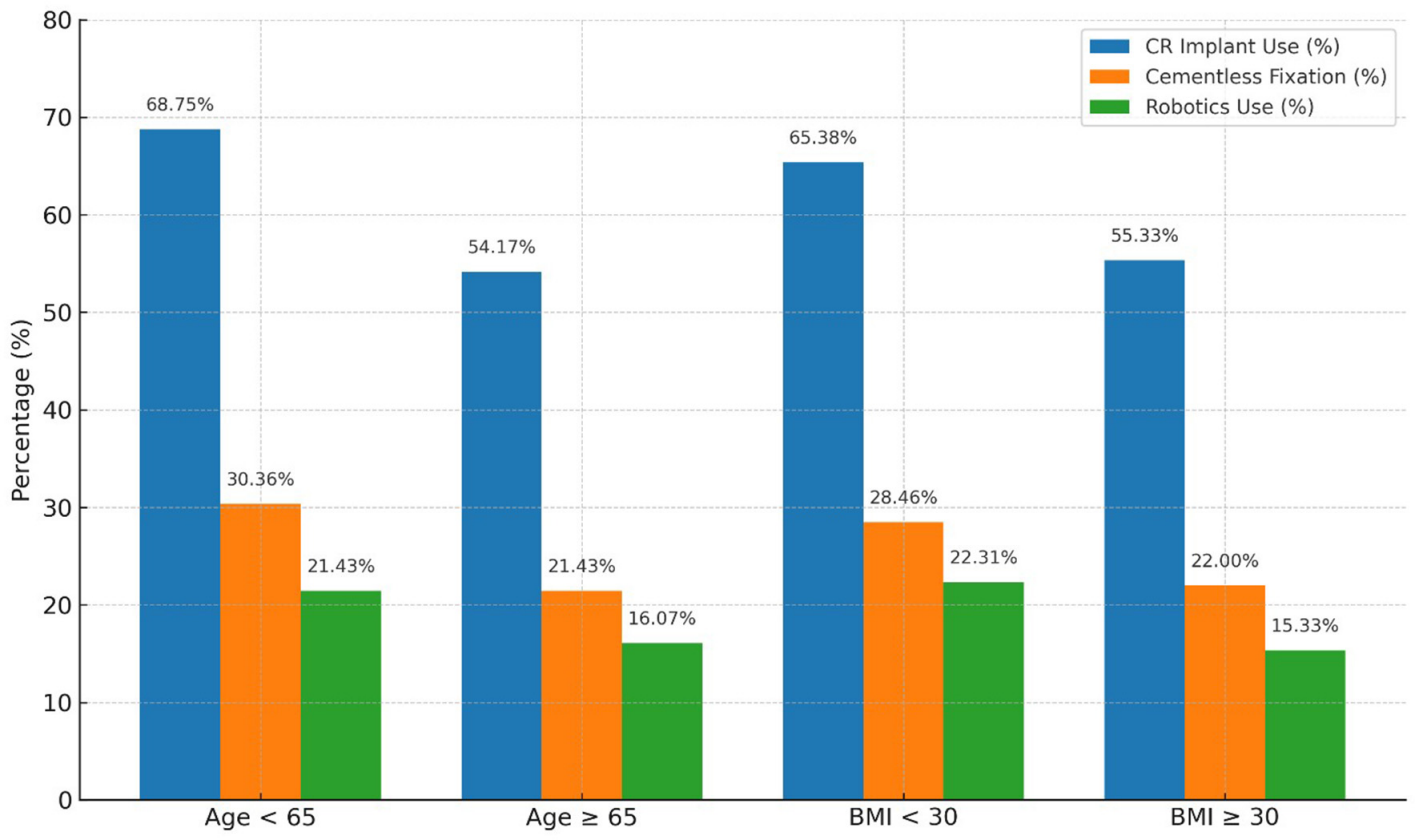
Sensitivity analysis: comparison of cruciate-retaining implant use, cementless fixation, and robotics utilization by age and BMI groups in total knee arthroplasty.

Sensitivity analysis revealed that patients younger than 65 years were more likely to receive cruciate-retaining implants than older patients (68.75% vs. 54.17%, *p* = 0.018) and had higher rates of cementless fixation (30.36% vs. 21.43%, *p* = 0.045) (Table 5 and Figure 3). Those in the younger age group also demonstrated greater use of robotic assistance (21.43% vs. 16.07%). When stratified by BMI, patients with BMI < 30 kg/m^2^ had higher CR implant use than those with BMI ≥ 30 kg/m^2^ (65.38% vs. 55.33%, *p* = 0.032). However, the difference in cementless fixation rates between BMI groups (28.46% vs. 22.00%) did not reach statistical significance (*p* = 0.061). Robotic utilization was consistently higher in both younger and lower-BMI patients, indicating a potential trend toward greater use of advanced technology in this subgroup.

**Table 5:** Sensitivity analysis: implant choice stratified by patient age and BMI.

Stratification group	Number of patients (*n*)	Mean age (years) ±SD	Mean BMI (kg/m^2^) ±SD	CR implant use (%)	PS implant use (%)	Cementless fixation (%)	Robotics use (%)	*p*-value (CR vs. PS)	*p*-value (cementless)
Age < 65 years	112	60.12 ± 3.45	27.80 ± 2.12	68.75%	31.25%	30.36%	21.43%	0.018	0.045
Age ≥65 years	168	71.20 ± 4.76	30.65 ± 3.21	54.17%	45.83%	21.43%	16.07%	0.018	0.045
BMI < 30 kg/m^2^	130	–	27.15 ± 1.92	65.38%	34.62%	28.46%	22.31%	0.032	0.061
BMI ≥30 kg/m^2^	150	–	32.88 ± 2.95	55.33%	44.67%	22.00%	15.33%	0.032	0.061

CR, cruciate-retaining; PS, posterior-stabilized; SD, standard deviation; BMI, body mass index; TKA, total knee arthroplasty.

## 4 Discussion

This cross-sectional study aimed to evaluate implant selection patterns in primary total knee arthroplasty and to identify surgeon- and patient-level factors associated with the use of cruciate-retaining vs. posterior-stabilized implants. The analysis demonstrated that significant variation in implant choice is associated with surgeon experience, fellowship training, and the use of robotic technology. Fellowship-trained and early-career surgeons suggested a greater preference for cruciate-retaining designs and were less likely to use cementless fixation. Multivariate analysis confirmed that specific surgeon characteristics and intraoperative technologies were independently associated with implant selection, whereas patient demographics, such as age and sex, also contributed. Sensitivity analyses further supported that younger and lower-BMI patients were more likely to receive cruciate-retaining implants and undergo robotic-assisted procedures, highlighting the interplay between patient characteristics and surgical decision-making. This study was conducted as a retrospective observational analytical study, and the findings were interpreted within a cross-sectional analytical framework.

The observed patterns in implant utilization are likely driven by established practice norms, clinical experience, and evolving surgical philosophies regarding knee kinematics and implant survivorship (12). The predominance of cruciate-retaining (CR) implant selection in this cohort is consistent with prior literature suggesting that CR designs may better preserve proprioception and replicate native knee biomechanics (7). Previous studies, such as those by Movassaghi et al. (4), have indicated that CR designs are frequently preferred in patients with intact posterior cruciate ligaments and minimal deformity, conditions common in our cohort. Steinert et al. (7) also reported improved long-term functional outcomes with CR designs in appropriately selected patients. Additionally, Green et al. (25) demonstrated comparable survivorship between CR and posterior-stabilized (PS) designs, suggesting that CR implants are a viable first-line option in routine cases. The higher rate of cemented fixation aligns with registry data from studies such as Lauck et al. (10), which continue to support cemented fixation as the gold standard for its predictable long-term outcomes. Although cementless TKA has gained interest, particularly among younger or high-demand patients, its broader adoption remains limited due to concerns regarding initial fixation reliability and long-term data, as highlighted by Alzarooni et al. (9). Although technology-assisted cases may have differed in case complexity or surgeon selection patterns, the current adjusted model suggested that robotic assistance, but not navigation, was independently associated with a higher likelihood of selecting a cruciate-retaining implant (26). However, non-random allocation of technology use may have introduced selection bias that was not fully controlled in the present analysis.

The association between implant choice and surgeon-level factors, such as fellowship training and years in practice, likely reflects differences in training exposure, familiarity with techniques, and alignment with evidence-based protocols, although causal relationships cannot be established (27). These findings should be interpreted strictly as associations rather than causal relationships. Accordingly, all identified relationships should be interpreted as associations rather than determinants of implant selection. These explanations remain interpretive and should not be construed as evidence of causality. All findings are reported as associations, and no direct inference regarding drivers of surgeon decision-making can be drawn from this analysis. The difference observed between unadjusted and adjusted analyses for years in practice further supports the influence of confounding. While unadjusted results suggested greater use of cruciate-retaining implants among surgeons with fewer years in practice, adjustment for key variables—such as age, institutional setting, and surgical volume—demonstrated that surgeons with ≥10 years in practice had higher odds of selecting these implants. Fellowship-trained surgeons may be more attuned to recent literature and evolving implant philosophies, which can influence decision-making patterns (27). Similar findings were reported by Zimnoch et al. (28), who noted that surgeons' training backgrounds significantly affected implant selection and the adoption of advanced technologies. Our results are also consistent with the work of Paul et al. (29), who found that institutional practice culture and surgeon-specific experience were major determinants of procedural variation in TKA. Furthermore, robotic-assisted procedures were more commonly employed by younger and fellowship-trained surgeons, consistent with the findings of Ali et al. (30), who observed that adoption of robotic technology is more prevalent among recently trained arthroplasty specialists. The influence of patient age and BMI on CR implant selection is consistent with the findings of Hickey et al. (31), who reported that younger, lower-BMI patients were more likely to meet anatomic criteria favorable for CR implant use. Together, these results underscore the importance of both surgeon characteristics and patient selection in guiding implant-related decisions during primary total knee arthroplasty (32).

Although higher BMI was associated with posterior-stabilized implant selection, the present analysis did not evaluate whether this association was modified by surgeon experience or whether greater surgeon confidence in managing technically complex cases contributed to this pattern. No interaction analysis between surgeon experience and BMI >30 was performed, and causation cannot be inferred from the current data. This may reflect multiple factors, including intraoperative exposure considerations, soft-tissue balancing demands, implant preference, or case complexity, rather than surgeon confidence alone (33). Future analyses incorporating interaction modeling may better clarify whether surgeon experience influences implant selection specifically in patients with obesity (33).

### 4.1 Clinical significance

This study offers clinically relevant insights into current selection patterns for implant designs in primary total knee arthroplasty at a high-volume tertiary center, highlighting how both surgeon-related factors and patient demographics influence the choice between cruciate-retaining and posterior-stabilized designs. The findings reinforce that fellowship training, years of practice, and the use of robotic technology are significantly associated with implant preference, emphasizing the role of advanced training and technological familiarity in shaping surgical decision-making. Additionally, the differential selection of implants based on patient age and BMI underscores the importance of personalized planning to optimize functional outcomes. These results may support the development of institutional guidelines to reduce practice variability and promote evidence-based implant selection strategies in total knee arthroplasty.

### 4.2 Limitations of the study and areas of future research

This single-center cross-sectional study, despite a robust sample size, was limited by its observational design and lack of longitudinal follow-up to assess clinical outcomes associated with specific implant types. Inclusion of only 10 surgeons may limit generalizability, and several potentially important confounding factors, including intraoperative findings, patient-reported preferences, intraoperative ligament quality, deformity severity, and radiographic parameters, were not captured and may have influenced implant selection. In addition, surgeon-level clustering was not accounted for using hierarchical modeling or random-effects methods, which may have affected variance estimation. Because surgeon-related variables were central to the analysis and the dataset included only 10 surgeons, standard logistic regression may have underestimated the uncertainty around surgeon-level associations due to within-surgeon correlation. Accordingly, these findings should be interpreted with caution. Future studies with larger surgeon samples should use mixed-effects logistic regression or generalized estimating equations to better account for clustering and obtain more robust variance estimates. Although the events-per-variable ratio was within commonly accepted thresholds, the number of events relative to the covariates included in the regression model remains a consideration when interpreting model stability and precision. Data on functional outcomes and long-term implant performance were also not assessed. Because technology use was not randomized, residual selection bias related to indication, surgeon preference, or case complexity may have remained. These factors should be considered when interpreting the reported associations. Future research should incorporate multicenter prospective designs with outcome measures to determine whether the identified implant selection patterns translate into differences in patient satisfaction, survivorship, or complication rates. Propensity score matching or other causal adjustment methods may further address selection bias in future analyses, and studies that explore standardized protocols across training backgrounds may help reduce surgeon-driven variability.

## 5 Conclusions

This study demonstrated that implant selection in primary total knee arthroplasty is significantly associated with surgeon experience, fellowship training, and intraoperative technology use, with cruciate-retaining implants more frequently selected by younger, fellowship-trained surgeons. Patient characteristics, such as age and BMI, were also associated with implant choice, particularly regarding cruciate-retaining design and cementless fixation. These findings highlight the multifactorial nature of implant decision-making and underscore the need for consistent, evidence-based approaches to reduce variability in clinical practice.

## Data Availability

The datasets presented in this study are publicly available in the online repository “Zenodo”. All versions of the dataset can be accessed and cited using the 10.5281/zenodo.18478951.
